# Plasma adrenomedullin is associated with short-term mortality and vasopressor requirement in patients admitted with sepsis

**DOI:** 10.1186/cc13731

**Published:** 2014-02-17

**Authors:** Rossella Marino, Joachim Struck, Alan S Maisel, Laura Magrini, Andreas Bergmann, Salvatore Di Somma

**Affiliations:** 1 University La Sapienza Rome, Sant’Andrea Hospital, Rome, Italy; 2 Adrenomed AG, Hennigsdorf, Germany; 3 Sphingotec GmbH, Hennigsdorf, Germany; 4 Veterans Affairs San Diego Healthcare System, San Diego, CA, USA

## Abstract

**Introduction:**

The incidence of death among patients admitted for severe sepsis or septic shock is high. Adrenomedullin (ADM) plays a central role in initiating the hyperdynamic response during the early stages of sepsis. Pilot studies indicate an association of plasma ADM with the severity of the disease. In the present study we utilized a novel sandwich immunoassay of bioactive plasma ADM in patients hospitalized with sepsis in order to assess the clinical utility.

**Methods:**

We enrolled 101 consecutive patients admitted to the emergency department with suspected sepsis in this study. Sepsis was defined by fulfillment of at least two systemic inflammatory response syndrome (SIRS) criteria plus clinical suspicion of infection. Plasma samples for ADM measurement were obtained on admission and for the next four days. The 28-day mortality rate was recorded.

**Results:**

ADM at admission was associated with severity of disease (correlation with Acute Physiology and Chronic Health Evaluation II (APACHE II) score: r = 0.46; *P* <0.0001). ADM was also associated with 28-day mortality (ADM median (IQR): survivors: 50 (31 to 77) pg/mL; non-survivors: 84 (48 to 232) pg/mL; *P* <0.001) and was independent from and additive to APACHE II (*P* = 0.02). Cox regression analysis revealed an additive value of serial measurement of ADM over baseline assessment for prediction of 28-day mortality (*P* < 0.01). ADM was negatively correlated with mean arterial pressure (r = -0.39; *P* <0.0001), and it strongly discriminated those patients requiring vasopressor therapy from the others (ADM median (IQR): no vasopressors 48 (32 to 75) pg/mL; with vasopressors 129 (83 to 264) pg/mL, *P* <0.0001).

**Conclusions:**

In patients admitted with sepsis, severe sepsis or septic shock plasma ADM is strongly associated with severity of disease, vasopressor requirement and 28-day mortality.

## Introduction

The global incidence of severe sepsis is greater than of either cancer or myocardial infarction, with a mortality rate estimated at 40% [1-3]. This high mortality is in many cases linked to multi-organ hypoperfusion and hypotension associated with the development of septic shock. As the clinical and laboratory findings of sepsis are nonspecific and culture results are not readily available, the diagnosis and risk stratification of patients is often delayed [4]. Biomarkers such as C-reactive protein (CRP), leukocyte count, lactate and Procalcitonin (PCT) are often used to differentiate between systemic inflammatory response syndrome (SIRS), sepsis, severe sepsis and septic shock as well as for patient risk stratification [5-7].

Adrenomedullin (ADM) is a peptide first isolated from a human pheochromocytoma [8], and has been found to be elevated in plasma of sepsis patients [9-11]. The mechanism of secretion of ADM in large part relates to the effects of lipopolysaccharide (LPS) stimulation [12]. Previous studies indicate that ADM plays a major role in initiating the hyperdynamic response during the early stages of polymicrobial sepsis [13,14]. Due to the cumbersome competitive immunoassays requiring extraction of large sample volumes, thus far plasma ADM has been determined only in a small number of sepsis patients [9,15-17]. Therefore, the potential value of determining plasma ADM in such patients cannot yet be ascertained. Recently, a new double-monoclonal antibody sandwich immunoassay for the measurement of human mature ADM has been developed facilitating the reliable and simple assessment of this biomarker [18]. The assay selectively detects the C-terminally amidated form of ADM, which in contrast to the glycine-extended ADM variant is bioactive. Our study aimed to explore the clinical utility of basal and serial plasma ADM assessments in patients hospitalized for sepsis using this new assay.

## Materials and methods

### Patient population

This was a prospective observational study performed in the Emergency Department (ED) Sant’Andrea Hospital University La Sapienza Rome, from December 2011 to April 2012. Patients presenting to the ED with suspected sepsis according to the Surviving Sepsis Campaign guidelines [19] and need for hospitalization were considered eligible for the study. We enrolled 101 consecutives patients (61 males and 40 females) with median (IQR) age 78 (72 to 83) years. Numerous comorbidities such as diabetes and hypertension were present: 29% of the patients had severe sepsis or septic shock on admission (Table 1).

**Table 1:** Patient characteristics

**Variable**	**Total population (n = 101)**
**Demographics**	
Gender, male, n (%)	61 (60.4)
Age, years, median (IQR)	78 (72 to 83)
**Examination variables**	
Respiratory rate, acts/minute, median (IQR)	24 (22 to 28)
Body temperature,°C, median (IQR)	38.4 (36 to 38.7)
Glasgow Coma Scale, points, median (IQR)	15 (10 to 15)
Mean arterial pressure, mm Hg, median (IQR)	83.3 (74 to 93)
**Past medical history**	
Cardiovascular, yes, n (%)	26 (25.7)
Hypertensive, yes, n (%)	47 (46.5)
Diabetes, yes, n (%)	35 (34.7)
Cancer, yes, n (%)	13 (12.9)
**Labaratory variables**	
ADM, pg/mL, median (IQR)	53.8 (37.4 to 94.0)
PCT, ng/mL, median (IQR)	2.8 (0.6 to 10.7)
Creatinine clearance, mL/minute, median (IQR)	48 (23 to 77)
Creatinine, mg/dL, median (IQR)	1.3 (0.9 to 2.4)
CRP, mg/dL, median (IQR)	16 (6.6 to 25.6)
White cells, 10^9^ cells/L, median (IQR)	12.7 (6.8 to 17.6)
Platelets, 10^9^ cells/L, median (IQR)	213 (150 to 278)
**Other**	
Final diagnosis, severe sepsis/shock, n (%)	29 (28.7)
Length of stay, days, median (IQR)	5 (2 to 7)
Septic shock at arrival, yes, n (%)	25 (24.8)
Acute organ dysfunction, yes, n (%)	39 (38.6)
APACHE II score, points, median (IQR)	16 (13 to 21)
**Treatment at baseline**	
Steroids, yes, n (%)	16 (15.8)
Vasopressors, yes, n (%)	18 (17.8)
Antibiotics, yes, n (%)	101 (100)
Fluid therapy, yes, n (%)	101 (100)

ADM, Adrenomedullin; PCT, Procalcitonin; CRP, C-reactive protein.

All patients gave informed written consent according to the Helsinki declaration. The study was approved by the ethical committee of Sant’ Andrea Hospital in Rome. Anamnestic data, physical examination, electrocardiogram, routine laboratory test, and instrumental radiologic examinations were performed for each patient. At arrival and for the first 4 days after hospitalization all patients underwent monitoring of vital parameters, including temperature, physical examination, arterial blood analysis, and fluid balance. The acute physiology and chronic health evaluation II (APACHE II) score was calculated during the first 24 hours after admission [20]. During hospitalization therapy, physical examination, fluid balance, vital parameters and culture examinations were recorded on a clinical research form. Survival was recorded daily by in-hospital observation and later by phone call, respectively, over a period of 28 days after admission to the ED.

### ADM measurement

Ethylenediaminetetraacetic acid (EDTA) plasma samples for measurement of ADM were obtained at arrival in ED and on the next 4 days during hospitalization. Aliquots for ADM measurement were stored at -40°C and then measured in a blinded fashion batch-wise using a recently developed immunoassay [18]: The ADM assay is a one-step sandwich-coated tube chemiluminescence immunoassay, based on Acridinium NHS-ester labeling for the detection of human ADM in unprocessed, neat plasma. It employs two mouse monoclonal antibodies, one directed against the mid region (solid phase), the other directed against the amidated C-terminal moiety of ADM (labeled antibody). Polystyrene tubes (Greiner Bio-One GmbH, Frickenhausen, Germany) were coated with the anti-mid-regional antibody (per tube 1.5 μg antibody in 300 μL of 50 mmol/L Tris, 100 mmol/L NaCl, pH 7.8) overnight at 22°C. Tubes were then blocked with 10 mmol/L Na-phosphate (pH 6.5) containing 3% Karion FP (Merck KGaA, Darmstadt, Germany) and 0.5% BSA (protease-free) and lyophilized. The anti-C-terminal antibody (1 g/L) was labeled by incubation with MACN-acridinium-NHS (N-hydroxysuccinimide)-ester (1 g/L; InVent GmbH, Hennigsdorf, Germany) in a 1:5 molar ratio for 20 minutes at 22°C. The reaction was stopped by addition of 1/5 volume of 50 mmol/L glycine for 10 minutes at 22°C. Labeled antibody was separated from free label by size exclusion chromatography first on a Centri Pure P5 Hydrated gel filtration column (emp Biotech GmbH, Berlin, Germany) and followed by a Bio-Silect® Sec 400–5 column (Bio-Rad Laboratories GmbH, München, Germany) for HPLC. For the immunoassay the labeled antibody was diluted into assay buffer (300 mmol/L K-phosphate; 100 mmol/L NaCl; 10 mmol/L sodium EDTA; 5 g/L BSA (protease-free) (Sigma); 1 g/L each of nonspecific bovine and mouse IgG; 0.9 g/L Na-azide; 20 tabs/L Protease Inhibitor Cocktail (Roche Diagnostics GmbH, Penzberg, Germany); 10 μmol/L Amastatin; 20 μmol/L Leupeptin; pH 7.0). Dilutions of full-length human ADM peptide (American Peptide Company, Sunnyvale, CA, USA) in Calibrator Dilution Buffer (10 mmol/L Tris; 250 mmol/L NaCl; 2 g/L Triton X-100; 50 g/L BSA (protease-free); 20 tabs/L Protease Inhibitor Cocktail (Roche AG); pH 7.0) served as calibrators. The immunoassay was performed as follows: 50 μL of plasma samples/calibrators and 200 μL of labeled detection antibody (800,000 relative light units (RLU) per 200 μL) were pipetted in the coated tubes. The assay was incubated for 18 hours at 4°C. Unbound tracer was removed by washing five times with wash solution (1 mL each). Tube-bound chemiluminescence was measured for 1 s using the LB953 Multi-Tube Luminometer (BERTHOLD TECHNOLOGIES GmbH & Co. KG, Bad Wildbad, Germany). The analytical assay sensitivity was 2 pg/mL. The median ADM concentration of 200 healthy subjects was 20.7 pg/mL; the 99th percentile was 43 pg/mL. The ADM sandwich assay used is not commercially available, but has been provided for research purposes by Sphingotec GmbH.

#### MR-proADM measurement

Admission EDTA plasma samples, which were available from 65 patients, were measured in the BRAHMS MR-proADM KRYPTOR immunoassay (Thermo Fisher Scientific, Hennigsdorf, Germany) according to the instructions of the manufacturer.

### Statistical analysis

Values are expressed as mean and standard deviation, median and IQR, or count and percentage as appropriate. The nonparametric Spearman correlation coefficient is given to describe the association between continuous variables. Group comparisons were performed using nonparametric tests (Kruskal-Wallis test for continuous variables and chi-square (χ^2^) test with simulated *P*-values using 2,000 replicates for categorical data). Biomarker data were log-transformed. To analyze the effect of individual risk factors on survival, univariable Cox proportional-hazards regression was calculated. The assumption of proportional hazard was tested for all variables. Log-transformed values of ADM and the APACHE score were evaluated in a multivariable Cox regression model to evaluate the contribution of ADM over and above that of APACHE alone based on nested models. The predictive value of each model was assessed by the model likelihood ratio chi-square statistic. The concordance index (C index) is given as an effect measure. Survival curves plotted by the Kaplan-Meier method were used for illustration. All statistical tests were two-tailed and a two-sided *P*-value of 0.05 was considered significant. The statistical analyses were performed using R version 2.5.1.

## Results

**Figure 1:**
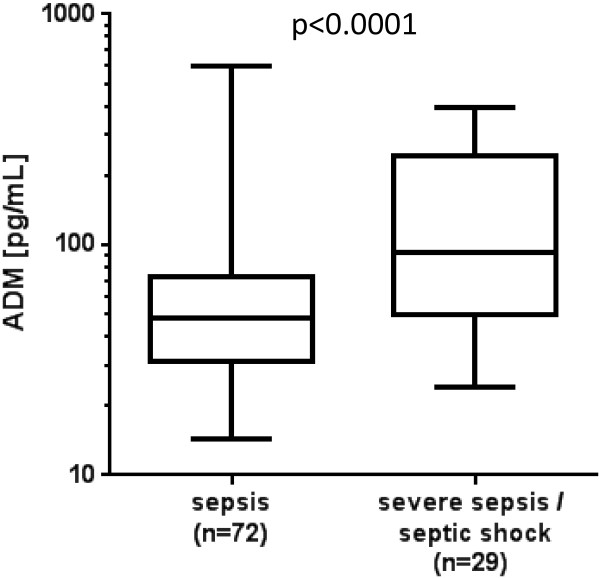
**Plasma Adrenomedullin (ADM) concentrations depending on severity of disease.** ADM concentrations are shown for patients with sepsis and severe sepsis/septic shock on admission.

At the time of admission, ADM concentrations were associated with the severity of disease: ADM was correlated with the APACHE II score (*r* = 0.46; *P* <0.0001), and were significantly (*P* <0.001) higher in patients presenting with severe sepsis or septic shock (93 (50 to 232) pg/mL) compared to those with sepsis only (48 (32 to 72) pg/mL, *P* <0.0001) (Figure 1).

**Figure 2:**
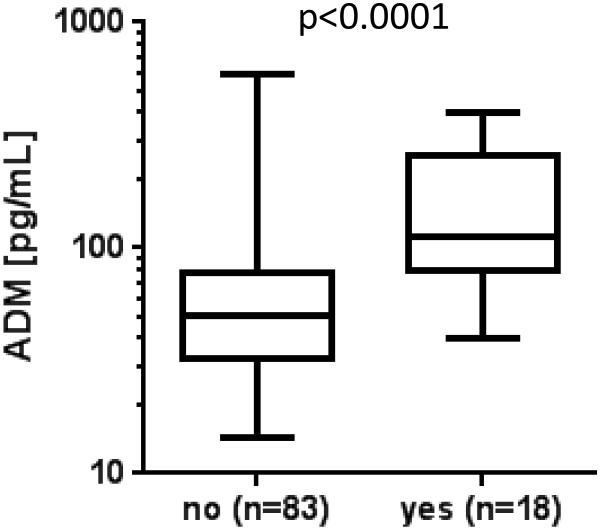
**Association of Adrenomedullin (ADM) with vasopressor treatment.** ADM concentrations are shown for patients who received (yes) or did not receive (no) vasopressor treatment on admission.

Admission ADM levels were negatively correlated with mean arterial pressure (MAP) (*r* = -0.39; *P* <0.0001). Patients who required vasopressors on admission had significantly (*P* <0.0001) higher ADM concentrations (129 (83 to 264) pg/mL) than those who did not require vasopressors infusion (48 (32 to 75) pg/mL) (Figure 2). There was a non-statistical trend toward higher ADM levels on admission (87 (44 to 133) pg/mL) in the four patients who later required vasopressor (87 (44 to 133) pg/mL) compared with those patients who did not receive vasopressor therapy during hospital stay (48 (32 to 72) pg/mL) (*P* = 0.448).

**Figure 3:**
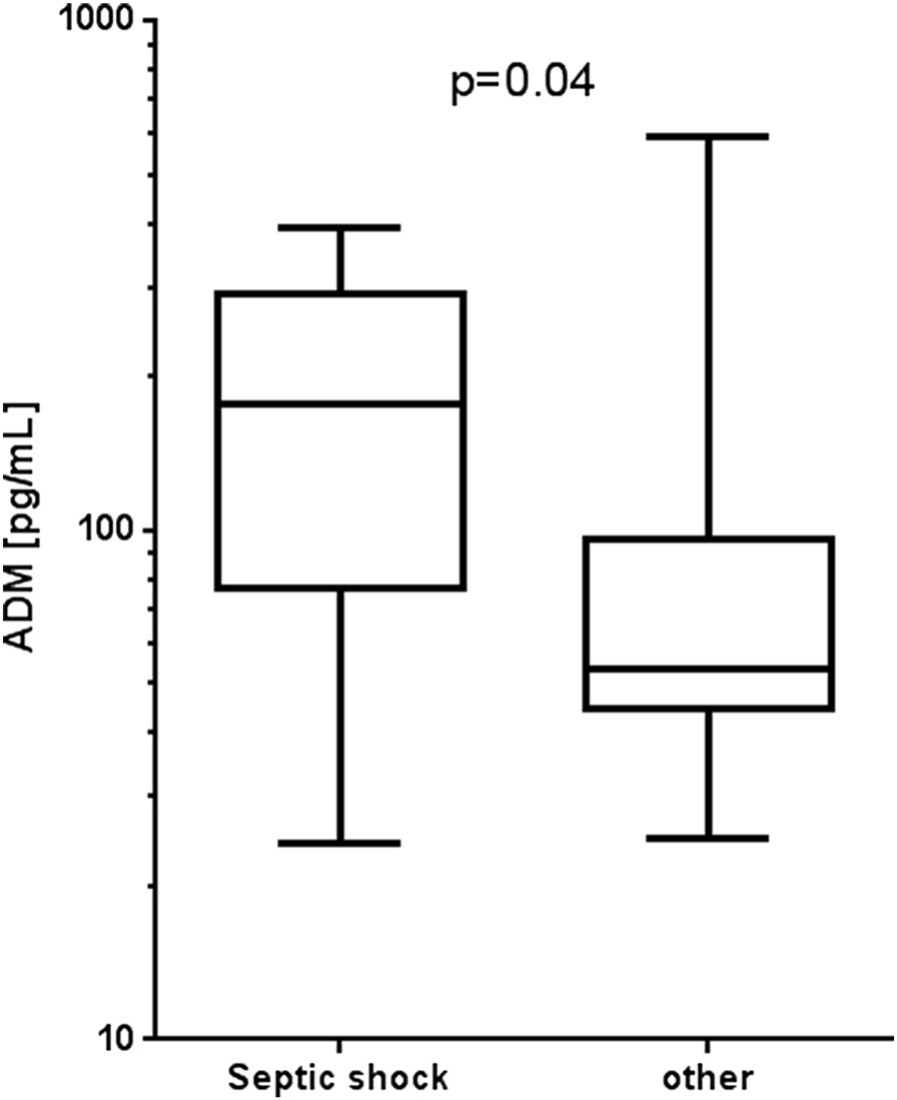
**Association of Adrenomedullin (ADM) with cause of death.** Admission ADM concentrations are shown for patients depending on their intra-hospital cause of death.

Over the observation period of 28 days following admission, 29 of the 101 patients died (28%). These patients had significantly higher admission ADM concentrations (84 (48 to 232) pg/mL) than the survivors (50 (31 to 77) pg/mL) (*P* <0.001) (Table 2). Median (IQR) of the APACHE II score was 22 (18 to 27) in non-survivors and 14 (12 to 18) in survivors (*P* <0.001). For the discrimination of survivors from non-survivors PCT and creatinine clearance were borderline significant (*P* = 0.0523 and *P* = 0.0539). Admission ADM levels were higher in patients who died in the hospital due to septic shock (177 (77 to 289) pg/mL) than in patients who died in the hospital due to other causes (54 (45 to 96) pg/mL) (*P* = 0.04) (Figure 3).

**Table 2:** Association of several variables obtained on admission with 28-day mortality

**Variable**	**Died within 28 days (n = 29)**	**Survived 28 days (n = 72)**	***P*-value**
ADM, pg/mL, median (IQR)	83.8 (48.3 to 232)	50 (31.2 to 77)	<0.001
PCT, ng/mL, median (IQR)	4.8 (1.4 to 13.9)	2.2 (0.6 to 8.9)	0.0523
Creatinine clearance, mL/minute, median (IQR)	31.5 (16 to 68)	56 (28 to 81)	0.0539
Creatinine, mg/dL, median (IQR)	1.8 (1 to 3.1)	1.25 (0.9 to 2.1)	0.0922
CRP, mg/dL, median (IQR)	17.4 (6.8 to 26.9)	14.5 (6.4 to 23.5)	0.7114
White cells, 10^9^ cells/L, median (IQR)	11.0 (26.5 to 17.2)	13.3 (8.1 to 17.7)	0.2416
Platelets, 10^9^ cells/L, median (IQR)	185 (127 to 237)	217 (162 to 299)	0.0979
APACHE II score, points, median (IQR)	22 (18 to 27)	14 (12 to 18)	<0.001

ADM, Adrenomedullin; PCT, Procalcitonin; CRP, C-reactive protein; APACHE II, acute physiology and chronic health evaluation II.

The standardized hazard ratio (HR) from the Cox regression model of continuous ADM levels at admission for predicting 28-day mortality was 2.6; (C index) area under the curve (AUC) = 0.69; χ^2^ = 18.2; *P* <0.0001. For the APACHE II score, the results from Cox regression analysis were (C index) AUC = 0.75; χ^2^ = 22.2; *P* <0.0001). ADM was independent from APACHE II and provided additional prognostic information (added χ^2^ = 5.2; *P* = 0.02). From a subset of 65 patients admission levels of MR-proADM were available. In the Cox regression model for predicting 28-day mortality MR-proADM performed worse ((C index) AUC = 0.60; χ^2^ = 1.46; *P* <0.22)) than ADM ((C index) AUC = 0.74; χ^2^ = 13.9; *P* <0.00019)).

**Figure 4:**
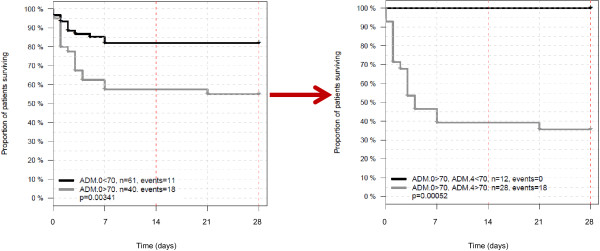
**Illustration of added value of serial Adrenomedullin (ADM) measurement.** Kaplan-Meier Plots for survival are shown for patients with ADM values above or below 70 pg/mL on admission (left) and for the subgroup of patients, who were above 70 pg/mL on admission and spread into those with ADM values above or below 70 pg/mL 4 days later (right).

We further assessed whether additional serial measurements of ADM in patients during their hospital stay would improve the prognostic value of ADM measurement on admission. The C index for predicting mortality continuously increased, the later ADM was measured (Table 3, *P* <0.01 for added value of ADM on days 3 and 4 on top of ADM on admission). The potential value of serial ADM measurement is illustrated in Figure 4: patients with an ADM admission concentration of above 70 pg/mL had a 28-day survival rate of 55%. For those patients from this group whose ADM concentration had remained above 70 pg/mL every day until 4 days after admission, the survival rate was 36%. In contrast, when ADM levels had decreased to concentrations below 70 pg/mL the survival rate was 100%.

**Table 3:** Cox regression analysis for prediction of 28-day mortality

**Variable**	**χ**^**2**^	***P*-value**	**C index (AUC)**	***P*-value of added value on top of ADM on admission**
ADM, admission	18.2	<0.0001	0.69	-
ADM, day 1	21.2	<0.0001	0.70	0.079
ADM, day 2	20.5	<0.0001	0.72	0.113
ADM, day 3	26.4	<0.0001	0.74	0.004
ADM, day 4	29.9	<0.0001	0.75	<0.001

If follow up data were missing, the last observation was carried forward. ADM, Adrenomedullin; AUC, area under the curve.

## Discussion

Using a novel ADM sandwich immunoassay, which selectively detects the bioactive C-terminally amidated ADM variant, this study demonstrates that plasma ADM levels in septic patients admitted to the ED increase with the severity of disease, correlate with the requirement for vasopressor therapy, and are associated with the 28-day mortality rate similar to APACHE II.

Although plasma ADM levels have been previously assessed in several small studies of septic patients [9,15-17], the assays used required large sample volumes, sample extraction, and long incubation times. Moreover, the technical validity of these competitive radioimmunoassays has been questioned [21]. Thus, there are restrictions with interpreting the ADM data of these studies due to the limited number of patients investigated and the type of ADM assays used, and consequently, the true potential clinical value of ADM measurement in septic patients has been difficult to judge. Measurement of a stable fragment of the ADM precursor peptide, namely MR-proADM, has been introduced to circumvent some of these existing problems [22-24]. However, conceptually, using MR-proADM as a surrogate for ADM has limitations: It does not reflect potential imbalances between the non-functional glycine-extended and the bioactive amidated ADM, and it must be assumed to have different clearing kinetics to ADM.

In the present study we demonstrated a strong association of admission ADM levels with the severity of the disease, supporting an earlier report describing elevated ADM levels in those with severe sepsis and those with septic shock [15]. It also supports several studies where MR-proADM was used as surrogate marker for ADM [7,23].

It is interesting that although ADM is known to be a potent vasodilator, in the present study it was negatively correlated to MAP, with a strong association to the need for vasopressor therapy. A correlation of plasma ADM in septic shock patients with the relaxation of vascular tone has been previously observed [17]. In our study, the patients who only later developed the need for vasopressor therapy actually had elevated ADM levels at admission. Clearly, a larger study is needed to confirm that elevation of ADM precedes the requirement for vasopressor therapy. If confirmed, measurement of ADM could become a helpful additional tool to guide vasopressor therapy.

In one study investigating only 12 septic patients, admission ADM did not distinguish survivors from non-survivors and did not correlate with mean arterial pressure [9]. Our much larger study with a more robust assay demonstrates that ADM levels on admission were strongly associated with the 28-day mortality rate. In fact, ADM was superior over PCT, creatinine clearance and, albeit tested only in a subset of patients, MR-proADM. Admission ADM levels were higher in those who died from septic shock in the hospital compared to other causes of death.

Using Cox regression analysis we could demonstrate that the accuracy for predicting mortality with plasma ADM levels increases, the later the ADM measured is made during the hospital stay. This indicates that serial measurement of ADM may add important information to admission levels and ADM might be suitable for therapy monitoring of sepsis patients. We illustrated the potential of monitoring using ADM by applying a cut off value of 70 pg/mL, which is close to the 99th percentile of the normal range (43 pg/mL): patients who were admitted with ADM levels above 70 pg/mL had a 28-day survival rate of 55%. For those patients from this group, whose ADM levels dropped to levels below 70 pg/mL 4 days later, the survival rate was 100%. The cut off was selected retrospectively as an example for how to distinguish groups with different risks to experience an adverse outcome depending on serial development of a biomarker concentration. The optimal cut off needs to be validated in future studies.

## Conclusions

In patients admitted to the ED with sepsis, severe sepsis, or septic shock, plasma ADM assessment is strongly associated with severity of disease, vasopressor requirement and 28-day mortality. Further research is warranted to assess in more depth the possible clinical utility of ADM measurement in patients hospitalized with sepsis.

## Key messages

• In sepsis patients admitted to the ED, mature plasma (ADM) measured by a novel sandwich immunoassay, is associated with severity of disease, 28-day mortality rate (independent from and additive to APACHE II), and requirement for vasopressor therapy.

• Serial measurement of ADM improves mortality risk prediction.

## Abbreviations

ADM: Adrenomedullin; APACHE: II acute physiology and chronic health evaluation II; BSA: bovine serum albumin; CRP: C-reactive protein; ED: Emergency Department; EDTA: ethylenediaminetetraacetic acid; HPLC: high-performance liquid chromatography; MAP: mean arterial pressure; PCT: Procalcitonin; SIRS: systemic inflammatory response syndrome.

## Competing interests

University La Sapienza Rome, Sant’Andrea Hospital, received a research grant from Sphingotec GmbH for this study. Andreas Bergmann is General Manager of Sphingotec GmbH and owns shares of Sphingotec GmbH. Sphingotec GmbH holds patent rights on the Adrenomedullin assay. Joachim Struck is employed by Sphingotec GmbH.

## Authors’ contributions

RM and LM recruited the patients, and obtained samples, clinical and laboratory variables. JS, RM, ASM and AB evaluated the data and drafted the manuscript. AB and SD conceived of the study, and participated in its design and coordination. All authors revised, read and approved the final manuscript.
